# Clinical care of family members of patients with dilated cardiomyopathy: A clinical consensus statement of the Heart Failure Association of the European Society of Cardiology, the European Society of Cardiology Working Group on Myocardial & Pericardial Diseases, and the European Society of Cardiology Council on Cardiovascular Genomics

**DOI:** 10.1093/eurheartj/ehaf571

**Published:** 2025-09-03

**Authors:** Job A J Verdonschot, Juan Pablo Kaski, Folkert W Asselbergs, Elijah R Behr, Philippe Charron, Dana Dawson, Kristina H Haugaa, Petr Kuchynka, Luis R Lopes, Andrea Mazzanti, Lorenzo Monserrat, Antonis Pantazis, Sanjay K Prasad, Heribert Schunkert, Petar M Seferovic, Mary N Sheppard, Gianfranco Sinagra, J Peter van Tintelen, Maria Teresa Tome Esteban, Stephane R B Heymans, Pablo Garcia-Pavia

**Affiliations:** Department of Clinical Genetics, Maastricht University Medical Center+, P. Debyelaan 25, 6229HX Maastricht, The Netherlands; Department of Cardiology, Cardiovascular Research Institute Maastricht, Maastricht University, Universiteitssingel 50, 6229 ER Maastricht, The Netherlands; Centre for Paediatric Inherited and Rare Cardiovascular Disease, Institute of Cardiovascular Science, University College London, London, UK; Centre for Inherited Cardiovascular Diseases, Great Ormond Street Hospital, London, UK; Department of Cardiology, Amsterdam Cardiovascular Sciences, Amsterdam University Medical Centre, University of Amsterdam, Amsterdam, The Netherlands; Institute of Health Informatics, University College London, London, UK; The National Institute for Health Research University College London Hospitals Biomedical Research Centre, University College London, London, UK; Cardiovascular and GEbonics Research Institute, City St. George’s, University of London, London, UK; Cardiovascular Clinical Academic Group, St. George’s University Hospitals NHS Foundation Trust, London, UK; Filière Nationale de Santé CARDIOGEN, Paris, France; Genetics and Cardiology Departments, APHP, Sorbonne Université, INSERM 1166, Institute of Cardiology and ICAN Institute for Cardiometabolism and Nutrition, Pitié-Salpêtrière Hospital, Paris, France; Aberdeen Cardiovascular and Diabetes Centre, University of Aberdeen, Aberdeen, UK; Department of Cardiology, Karolinska University Hospital, Stockholm, Sweden; Department of Cardiology, Oslo University Hospital, Oslo, Norway; 2nd Department of Medicine, Department of Cardiovascular Medicine, First Faculty of Medicine, Charles University and General University Hospital, Prague, Czech Republic; Barts Heart Centre, St Bartholomew’s Hospital, London, UK; Institute of Cardiovascular Science, University College London, London, UK; Molecular Cardiology Unit, IRCCS Istituti Clinici Scientifici Maugeri, Pavia, Italy; Department of Molecular Medicine, University of Pavia, Pavia, Italy; Medical Department, Dilemma Solutions, A Coruña, Spain; Royal Brompton and Harefield Hospitals, London, UK; Royal Brompton and Harefield Hospitals, London, UK; Department of Cardiology, Deutsches Herzzentrum München, Universitätsklinikum der Technischen Universität München, Munich, Germany; Deutsches Zentrum für Herz- und Kreislauferkrankungen (DZHK), Partner Site Munich Heart Alliance, Munich, Germany; Department of Cardiology, Serbian Academy of Sciences and Arts and Faculty of Medicine, University of Belgrade, Belgrade, Serbia; CRY Cardiovascular Pathology Unit, Cardiovascular and Genetic Research Institute, St George’s, University of London, London, UK; Cardiothoracovascular Department, Azienda Sanitaria Universitaria Giuliano Isontina, Center for Cardiomyopathies, University of Trieste, Trieste, Italy; Department of Genetics, University Medical Center Utrecht, Utrecht, The Netherlands; Cardiovascular and GEbonics Research Institute, City St. George’s, University of London, London, UK; Cardiovascular Clinical Academic Group, St. George’s University Hospitals NHS Foundation Trust, London, UK; Department of Cardiology, Cardiovascular Research Institute Maastricht, Maastricht University, Universiteitssingel 50, 6229 ER Maastricht, The Netherlands; Department of Cardiovascular Sciences, Centre for Molecular and Vascular Biology, University of Leuven, Belgium; Heart Failure and Inherited Cardiac Diseases Unit, Department of Cardiology, Hospital Universitario Puerta de Hierro IDIPHISA, Manuel de Falla, 2, Madrid 28222, Spain; CIBER Cardiovascular Instituto de Salud Carlos III, Av. Monforte de Lemos, 3-5 28029 Madrid, Spain; Centro Nacional de Investigaciones Cardiovasculares (CNIC), Melchor Fernández Almagro 3, 28029 Madrid, Spain

**Keywords:** Dilated cardiomyopathy, Genetics, Screening, Family members

## Abstract

Genetic family screening following the detection of a pathogenic or likely pathogenic variant in a proband with dilated cardiomyopathy (DCM) remains one of the main applications of genetic testing. While cardiac screening is recommended for all first-degree relatives, the *a priori* risk among family members varies. Consequently, screening regimens should be tailored according to both genetic and clinical information at the individual and familial level. This clinical consensus statement provides tools to help with the risk assessment and follow-up of screening for family members and discusses the utility for integration of genotype-specific information, cardiac imaging, and electrocardiogram findings to personalize cardiac screening regimens, which in conjunction will likely improve individualized risk prediction. Early phenotypic detection of DCM in family members remains an active area of research and innovation. In addition, data are starting to accrue on the utility of early therapeutic intervention in family members with very mild phenotypes that may inform future management in addition to screening. A systematic strategy is proposed to determine the *a priori* risk of developing DCM for a family member, and the potential of integrating genotype–phenotype knowledge towards family management. Lastly, there is a focus on the current knowledge gaps and ongoing and future opportunities to improve risk prediction, early disease detection, and treatment of family members of patients with DCM.

## Graphical Abstract

**Graphical Abstract:**
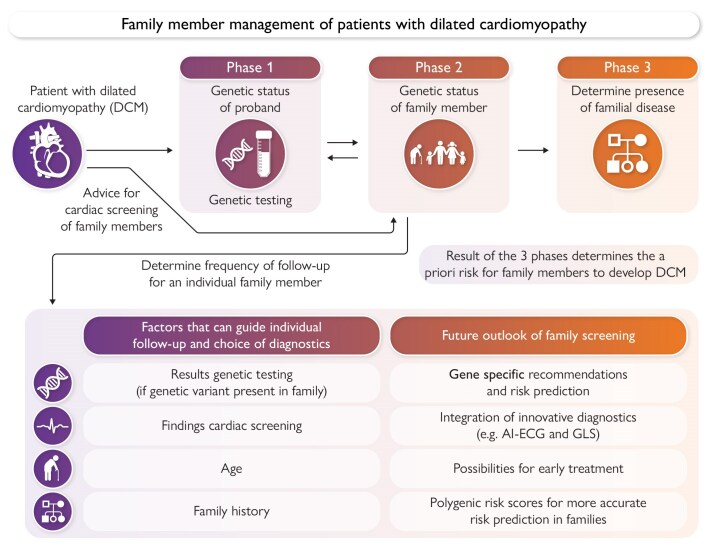
The results of genetic testing of the proband determines the options for a family member (phase 1). When a (likely) pathogenic variant is detected, the family member can choose to test for this variant (phase 2). In the absence of a (likely) pathogenic variant in the family, the risk for the family member to develop dilated cardiomyopathy can increase if multiple family members have the disease (phase 3). The results of phase 1-3 determines the risk for the family member to develop dilated cardiomyopathy, and can help to guide the cardiac follow-up. AI-ECG, artificial intelligence-guided electrocardiography; DCM, dilated cardiomyopathy; GLS, global longitudinal strain.

## Introduction

Genetic testing is a key aspect of the diagnostic work-up of patients with dilated cardiomyopathy (DCM).^1–4^ Besides the clinical consequences of a (likely) pathogenic variant for the patient, it also provides an opportunity to perform cascade screening with subsequent risk prediction on phenotype development for first-degree family members. Accurate and timely family assessment allows detection of DCM at an early stage or even at a preclinical stage in family members, creating a possible ‘window’ to prevent or delay irreversible cardiac injury, heart failure hospitalizations, and sudden cardiac death. The aim of this clinical consensus statement is to (i) provide tools for risk estimation of phenotype development for family members, (ii) integrate the available genotype–phenotype knowledge towards defining the most appropriate screening protocols, and (iii) describe the current knowledge gaps and explore the future opportunities to improve therapy and risk prediction of family members.

The focus of the current consensus statement is primarily on family members of patients with DCM. However, it should be acknowledged that the clinical spectrum of cardiomyopathies is broader than just the phenotype presenting with left ventricular (LV) dilatation and systolic dysfunction. The 2023 European Society of Cardiology (ESC) cardiomyopathy guidelines address this by including descriptions of the phenotype at presentation as the starting point for a diagnostic approach that aims to define a final diagnosis combining phenotype and underlying aetiology. In this context, non-dilated LV cardiomyopathy (NDLVC), characterized by LV scarring or fatty replacement or global LV hypokinesia in the absence of LV dilatation,^1^ shows genetic and phenotypic overlap with DCM and includes phenotypes previously included under the umbrella term ‘arrhythmogenic cardiomyopathy’ (ACM). Even within the same family with a specific genetic variant, the phenotype can differ per individual (e.g. a FLNC variant can lead to DCM, NDLVC, or a more arrhythmogenic phenotype within the same family). It is therefore important to acknowledge the spectrum of phenotypes associated with specific genotypes and to recognize these while screening asymptomatic family members.

## Definition and categorization of family members at risk

**Figure 1:**
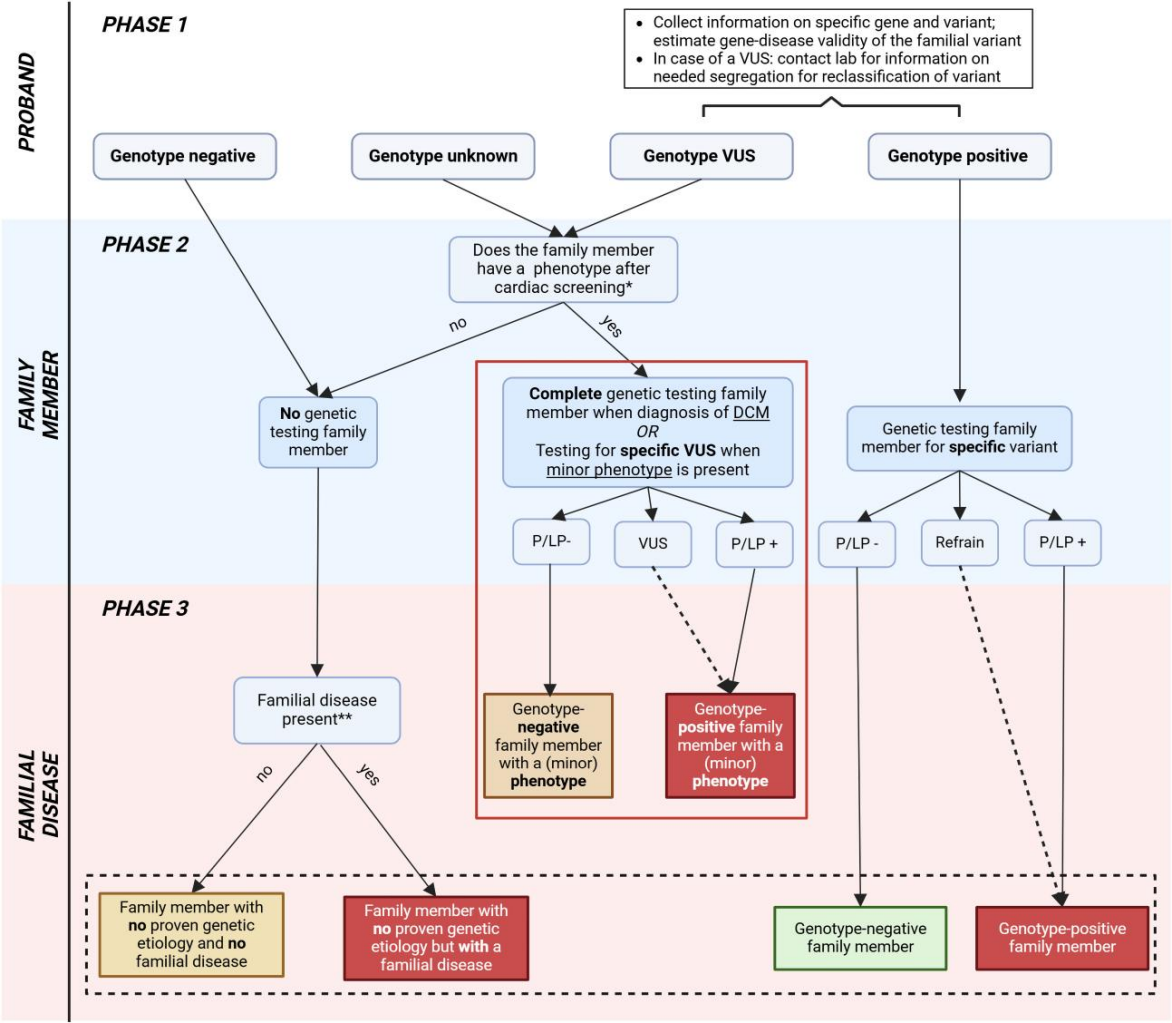
Characterizing the genetic status of a family with dilated cardiomyopathy. The dashed line at the variant of unknown significance indicates that after cardiac screening and segregation, the variant of unknown significance could be reclassified towards (likely) pathogenic. The dashed line at refrain indicates that family members that refrain from genetic testing for the familial variant should be considered as genotype-positive family members until they are tested for the familial variant. The box with the solid line contains family members with a (mild) phenotype whose follow-up will deviate from unaffected family members, as they will be followed based on their phenotype. The box with the dashed line indicates risk categories of family member without phenotype; see *Figure 2* for the follow-up of these individuals. *If the proband has a variant of unknown significance or the genotype is unknown, cardiac screening of the first-degree family members is advised before potential genetic testing. When a (minor) phenotype is detected (*Table 1*), genetic testing of the affected family member can be considered. **Definition of familial disease: two or more individuals (first- or second-degree family members) in a single family who are diagnosed with dilated cardiomyopathy, or a proband with dilated cardiomyopathy and a first-degree family member with autopsy-proven dilated cardiomyopathy or sudden death below the age of 50 years

Dilated cardiomyopathy is defined by the presence of LV dilatation (LV dimensions or volumes > 2 z-scores above population mean values corrected for body size, sex, and/or age) and systolic dysfunction [LV ejection fraction (LVEF) < 50%] unexplained solely by abnormal loading conditions or coronary artery disease.^1^ Genetic testing is recommended in all patients fulfilling the criteria for DCM as it enables cascade genetic evaluation of family members according to the 2023 ESC guidelines for the management of cardiomyopathies^1^. Estimating the *a priori* risk of a family member is the first step in the diagnostic work-up, which is based on the genetic status of the proband (i.e. the first patient in a family who is diagnosed with DCM), and the results of cardiac screening and genetic testing of the family member. The *a priori* risk is of great importance in the further management and can be established by evaluating three phases (*Figure 1* and *Graphical Abstract*).

### Phase 1: determining the genetic status of the proband

Genetic information of the affected proband is necessary to estimate the risk for the family member, and considerable effort should be made to obtain this information. In genetic testing of the proband, three scenarios can be encountered: (i) a pathogenic/likely pathogenic (P/LP) variant could be identified in the proband (i.e. genotype positive), (ii) no P/LP variant (i.e. genotype negative), or (iii) a variant of unknown significance (VUS) has been identified. Not infrequently, genetic testing may not have been performed or declined in the proband, and their genetic status remains unknown.

If a P/LP variant is found, it is paramount to check whether the affected gene has a valid gene–disease association (i.e. whether variants in the gene can lead to DCM). Efforts to systematically curate evidence for the clinical validity of gene–disease relationships associated with DCM have had an important impact on narrowing gene lists, including work by the Clinical Genome Resource (ClinGen) gene curation expert panel (www.clinicalgenome.org)^5–7^. These need to be checked when estimating the clinical relevance of a detected variant. Many of these contributors have come together in an international coalition (the Gene Curation Coalition; thegencc.org) to share, standardize, and disseminate this information for the community.

### Phase 2: determining the genetic status of the family member

If a P/LP variant is detected in the proband, pre-symptomatic testing of the specific variant can be offered to (asymptomatic) first-degree family members after counselling. Exact information about the gene and variant in the family is important, to ensure that the correct variant is tested and that there is an established gene–disease relationship. It is recommended by the 2023 ESC cardiomyopathy guideline that every family member that considers genetic testing receives pre- and post-test counselling by a trained individual with expertise.^1,4^ The family member also has the possibility to refrain from genetic testing. In that case, he or she should remain under cardiac screening surveillance until the absence of the familial variant is proven in the future. Common considerations for genetic testing of a familial P/LP variant include (but are not limited to) preventing unnecessary cardiac screening when they do not carry the variant, determining the (genetic) risk for their children, or reproductive possibilities when they do carry the P/LP variant.

When a VUS has been identified in the proband, family members are not advised to undergo genetic testing in the absence of a phenotype after cardiac evaluation. This is due to the fact that the causality of a VUS and the expression of DCM are unclear and therefore might cause unnecessary worry. If a family member has a clear DCM phenotype and genetic testing was not performed in the proband or a VUS was detected, complete genetic testing should be discussed with the affected family member (i.e. a panel containing at least the genes with a strong gene–disease association including the gene in which the VUS was found^5^) as the presence of a second P/LP variant in the family is possible and there is a separate indication for genetic testing, depending on the diagnostic possibilities. However, if the gene–phenotype correlation is very strong, it can also be considered to test for the specific VUS. Segregation (i.e. testing family members on a specific variant in combination with information on their phenotype) of a VUS among affected family members can provide valuable information for reclassification of the pathogenicity of the variant, thereby providing clarity of this specific variant for the family. However, segregation among a large number of kindreds is necessary to eventually support the pathogenicity of a variant, which can be calculated through the logarithm of the odds (LOD) score.^8,9^ Classically, a LOD score of >3 (e.g. a chance of 1000:1 that the variant is associated with disease) has been considered necessary to support pathogenicity of a variant, but we recommend contacting the diagnostic genetic laboratory to determine whether segregation is achievable in a family for reclassification of a specific VUS. However, segregation is challenging and often families are not large enough to achieve reclassification. Regular re-evaluation of the pathogenicity of a VUS is important, as this can change over time having clinical implications for that patient and their family. Therefore, care of genetic patients in a specialized centre with a procedure for VUS re-evaluation seems appropriate.^10^

### Phase 3: determining the presence of familial disease

Distinguishing between sporadic and familial diseases is important when the proband does not have a P/LP variant, or when genetic testing has not been performed. The definition of familial disease in the absence of a known genetic cause is the presence of:

1. Two or more individuals (first- or second-degree family members) in a family who are diagnosed with DCM
2. A proband with DCM and a first-degree family member with autopsy-proven DCM or sudden (cardiac) death below the age of 50 years

Healthcare professionals should always be aware that not fulfilling the criteria for familial disease does not exclude the possibility of a familial or genetic component.^2^ Asymptomatic family members could have DCM without being diagnosed (yet). In addition, cardiac screening of family members could reveal mild non-diagnostic abnormalities that overlap with normal variation, but could also be early signs of an unveiling DCM.^11^ See *Table 1* for mild abnormalities that deserve attention when detected in family members during cardiac evaluation.^3^

**Table 1:** Minor abnormalities suggestive of disease onset in family members

Electrocardiography
Complete left bundle branch block
AV block (PR > 200 ms or higher degree AV block)
Unexplained ventricular arrhythmia (>100 ventricular premature beats per hour in 24 h or non-sustained ventricular tachycardia, defined as ≥3 beats at a rate of ≥120 b.p.m.)
Atrial fibrillation or atrial flutter
Low voltage in the peripheral leads^a^
T-wave inversion in ≥2 contiguous leads
Echocardiography/CMR
Segmental wall motion abnormalities in the left ventricle in the absence of intraventricular conduction defect
Left ventricular dilatation^b^
Mildly decreased left ventricular ejection fraction
Late gadolinium enhancement of non-ischaemic pattern

AV, atrioventricular; CMR, cardiac magnetic resonance.

^a^Defined as a QRS amplitude of 5 mm (0.5 mV).

^b^Defined as ≥2 SD from those predicted according to body surface area and sex.

After going through these three phases, different categories of family members can be identified (*Figure 1*), which is important for estimating the *a priori* risk for DCM, determining cardiac evaluation and a subsequent prospective screening regimen.

## Genetic counselling and cardiac screening

Pre- and post-test counselling by a trained individual with expertise is recommended for every first-degree family member, as detailed in the 2023 ESC guidelines for the management of cardiomyopathies.^1^ When a first-degree family member of a patient with DCM is deceased, it is important to consider counselling of close relatives (e.g. second-degree family members of the proband). Important concepts that need to be discussed with an asymptomatic family member can be found in *Table 2*. Genetic counselling can improve knowledge, recall, and patient empowerment, but also increase satisfaction with decision-making.^12,13^ It is a process that aims to support patients and their families to understand and adapt to the medical, psychosocial, and familial impact of genetic diseases.

**Table 2:** Key discussion points of pre- and post-test (genetic) counselling of asymptomatic family members

Pre-test (genetic) counselling	Genetic education and mode of inheritance (possible implication for children): discuss available familial information about genetic variant and family history and retrieve additional needed information where possible
	Process and logistics of genetic testing and return of the result
	Impact of genetic testing on insurance^a^
	Implications for clinical care and cardiac screening and follow-up
	Reproductive options for P/LP variant carriers (options differ per country)
	Possibility to refrain from genetic testing (but choice for cardiac screening instead)
	Explanation of possible outcomes (P/LP present or absent)
	Psychosocial support
	*In case of a VUS in family*: possibility for segregation when this is deemed to be informative by the genetic lab (possibility for reclassification of variant)
	*In case of cardiac screening without genetic testing* ^b^: risk for developing DCM for family member (based on *a priori* risk) and option for cardiac screening and follow-up
Post-test (genetic) counselling	Result disclosure
	If P/LP variant not present: discharge from cardiac screening, return when new cardiac symptoms develop
	If P/LP variant present: implications for screening and follow-up (and reproductive options when applicable), implications for first-degree family members and how to inform them, exploration of feelings and understanding, offer psychosocial support

DCM, dilated cardiomyopathy; P/LP, pathogenic/likely pathogenic; VUS, variant of unknown significance.

^a^The insurance system differs per country, meaning that the consideration of genetic testing results in insurance decision-making is also different. In most countries, it is unlikely that insurance applications will be rejected, but a higher cost for specific insurances (e.g. life insurance) may be a consequence.

^b^For example, for family members of patients with dilated cardiomyopathy without a pathogenic/likely pathogenic variant or when genetic testing is not performed in the family. *A priori* risk based on *Figure 1*.

### Choice of diagnostics for screening of family members

**Figure 2:**
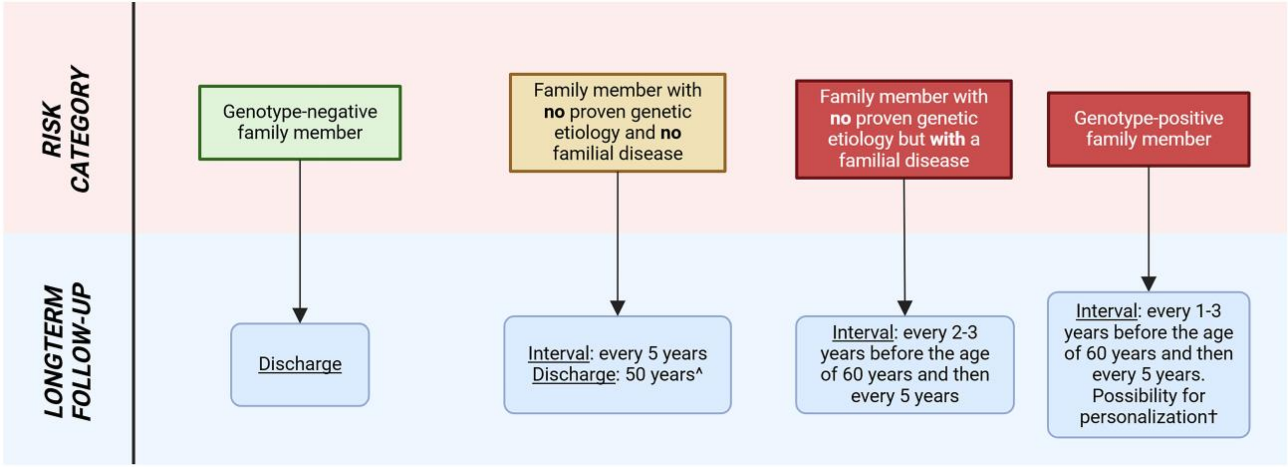
Screening and long-term follow-up of family members based on their *a priori* risk to develop dilated cardiomyopathy. Clinical screening is indicated for every first-degree family member of a patient with dilated cardiomyopathy according to the 2023 European Society of Cardiology guidelines on the management of cardiomyopathies^1^. ^Consider termination of periodic screening at the age of 50 years based on clinical information of the proband (e.g. presence of other non-ischaemic aetiologies of dilated cardiomyopathy and age of diagnosis in proband) in an individual with normal cardiac investigations. †See *Table 5* for further details

Based on the *a priori* risk, different screening strategies should be discussed with the family member (*Figure 2*). Baseline clinical screening including at least an electrocardiogram (ECG) and cardiac imaging is advised for every first-degree family member of a patient with DCM, irrespective of the *a priori* risk. Additional diagnostics can be guided by the age, initial findings on ECG and cardiac imaging, and dominant phenotype and genotype in the family (e.g. Holter analysis in the presence of a P/LP variant associated with a high risk for arrhythmias, or when arrhythmias are the dominant phenotype in the family). The latest ESC guideline on cardiomyopathies recommends echo and/or cardiac magnetic resonance (CMR) imaging for screening of family members.^1^ Information on the affected gene in the family can help guide the decision-making for the choice of imaging modality (*Table 3*). For example, P/LP variants in *FLNC*, *DSP*, and *PLN* can lead to cardiac fibrosis prior to signs of systolic dysfunction, while variants in *LMNA* can lead to premature conduction disease and atrial arrhythmias before dilation and systolic dysfunction.^14–18^ Furthermore, based on emerging data that recognize the presence of late gadolinium enhancement (LGE) on CMR as a feature strongly associated with DCM development, we recommend that symptomatic genotype-positive family members should be considered for screening with CMR at initial evaluation, especially those carrying a P/LP variant in a ‘high-risk’ gene (e.g. *LMNA*, *FLNCtv*, *DES*, *DSP*, *PLN*, *RBM20*, and *TMEM43*).^19^

**Table 3:** Genotype–phenotype associations of eight genes that are definitively associated with dilated cardiomyopathy according to the ClinGen consortium, which were investigated in large multi-centre cohorts

	Average age of disease onset (year)	Arrhythmias	CMR-LGE^20^	Sex differences	Penetrance	Outcome
Titin (*TTN*tv)^21^	40–50	Prevalent AF and NSVTs	Unspecific LGE pattern, mostly mid-wall in the septum	Disease penetrance higher in males	80% at age of 60	Variable from mild to severe
Lamin A/c (*LMNA*)^22^	30–40	Prevalent AF, NSVTs, and AVB	Unspecific LGE pattern	Higher risk of arrhythmias in males	100% at age of 60	High risk of HTx
Beta myosin heavy chain (*MYH7*)^23^	30–40^a24^		Rare		80% at age of 50	Low rate of LVRR and predominant HF-related events
Desmoplakin (*DSP*)^25^	30–40	Prevalent NSVTs and PVCs	Subepicardial, ring-like pattern	Disease penetrance and risk of arrhythmias higher in females		Aggressive clinical course; predominance of arrhythmic events
Phospholamban (*PLN*)^26^	40–50	Prevalent NSVTs and PVCs	Predominant LV inferolateral wall			Aggressive clinical course; predominance of arrhythmic events
BAG cochaperone 3 (*BAG3*)^27^	30–40	-	Unspecific LGE pattern	Worse outcome in males	80% at age of 40	Aggressive clinical course; predominance of HF-related events
RNA-binding motif protein 20 (*RBM20*)^28^	30–50^b^	Prevalent NSVTs	Rare	Disease onset at younger age in males^b^	80% at age of 45	Aggressive clinical course; predominance of arrhythmic events
Filamin C (*FLNCtv*)^29^	40–50	Prevalent NSVTs and PVCs	Subepicardial, ring-like pattern		80% at age of 40	Aggressive clinical course; predominance of arrhythmic events

AF, atrial fibrillation; CMR, cardiac magnetic resonance; DCM, dilated cardiomyopathy; NSVT, non-sustained ventricular tachycardia; AVB, atrioventricular block; LGE, late gadolinium enhancement; HF, heart failure; HTx, heart transplantation; LVRR, left ventricular reverse remodelling; PVC, premature ventricular contraction; HF, heart failure; tv, truncating variant.

^a^For *MYH7* variants, there is a high risk of onset of dilated cardiomyopathy before the age of 12 years.

^b^Age of onset significantly differs between males and females: males on average between 30 and 40 and females between 40 and 50.

### Predictors of phenotype development in family members

Evidence on the yield of cardiac screening of family members is mostly available through retrospective cohort studies, with variable population characteristics and variable definitions of (minor) phenotypes. Consequently, the reported yield of DCM at initial baseline evaluation of family members varies from 9% to 22%.^19,30–33^ The yield is the highest in family members carrying a P/LP variant, but also family members of gene-elusive probands could be diagnosed with DCM at initial baseline evaluation (*Table 4*). The incidence rate of DCM during serial cardiac screening is estimated to be around 2% per person-year in the overall DCM population, up to 8.9% per person-year in family members carrying a P/LP variant.^31^ In family members with a P/LP variant who have no apparent cardiac abnormalities at baseline screening, 11% develop DCM during a median follow-up of 3 years.^19^ The disease penetrance is likely to be higher, as data on longer follow-up duration are absent.

**Table 4:** Risk of developing dilated cardiomyopathy for family members based on the *a priori* risk

*A priori* risk family member		Prevalence of a phenotype at first screening^a^	Penetrance during short-term follow-up^b^
Status proband	Status family member		
Familial+	Not tested	0.6%^30^	NA
Genotype+	Genotype−	0%^31^	0%^31^
Genotype+	Genotype+	4%^30^	11%^19c^
Genotype unknown or VUS	Not tested	3%^30^	8%^31^

Data gathered from the few available publications. True disease penetrance is higher than reported in the few available publications on which this table is based, due to the lack of data of long-term follow-up.

NA, not available; VUS, variant of unknown significance.

^a^Prevalence reflects one moment in time where the age at first screening differs. Prevalence is age-dependent and is higher with increasing age.

^b^Penetrance is dependent on the duration of follow-up, which differed among studies (varying from 3 to 6 years). The numbers in the table are likely to be an underestimation of the true disease penetrance, as duration of follow-up in published studies is limited.

^c^Number reflects the penetrance of all genotype-positive individuals combined. The penetrance significantly differs per specific genotype.

Clinical predictors for the development of DCM in family members with a P/LP variant according to a recent study included age, abnormal ECG findings (i.e. negative T-waves in ≥2 contiguous leads, bundle branch block, atrial fibrillation, atrial flutter, or second-degree atrioventricular block), the presence of a P/LP variant in a motor sarcomeric gene, lower LVEF, larger LV end-diastolic diameter (both within the normal range), and LGE on CMR.^19^ Although these parameters still require external validation, they can be used to guide clinical screening and frequency of follow-up in those individuals at higher risk of developing DCM.

### Cardiac screening and follow-up of family members

In the presence of a familial P/LP variant, first-degree family members can be tested for the specific variant (i.e. cascade screening) and discharged from clinical follow-up if they do not carry the P/LP variant and do not have a phenotype. In this situation, they should be advised that their risk of developing DCM is the same as the general population and that they should seek medical advice if they develop new cardiac symptoms. In all other categories, cardiac follow-up of family members is appropriate (*Figure 2*).

In families with only one patient with DCM and no identified P/LP variant after comprehensive genetic testing and family screening (i.e. sporadic form of DCM), clinical follow-up of family members should start at an adult age with a screening interval of 5 years. Periodic screening could be terminated in informative families (i.e. families that are large enough with information about age of onset in the proband) from the age of 50 in family members with normal cardiac investigations. However, family members should always seek advice when they develop symptoms. In family members with demonstration of a familial disease and negative genetic testing, the proposed frequency is once every 2–3 years before the age of 60, and then every 5 years.^1^

For family members that carry a P/LP variant, the screening interval depends on the findings on baseline screening, genotype, age, and family history.^19^  *Table 5* provides guidance (and clinical examples) on how these parameters can help to estimate the screening interval for a specific individual. For example, a young patient who carries a P/LP variant in a high-risk gene (*FLNC*) with no abnormalities at baseline cardiac screening but with a family history of sudden cardiac death at 28 years should undergo annual cardiac screening. Screening with CMR during follow-up can be considered every 3–5 years, especially in those genes with a high risk for arrhythmic events (*Figure 2*; *Table 3*). Age for start of clinical screening of a family member should be based on the expected age of onset (taking into account certain genes with frequent paediatric onset such as *MYH7*), presentation in the family, and clinical/legal consequences. Currently, many centres start clinical cardiac screening in young adolescence. Start of cardiac screening and genetic screening in a paediatric relative should be based on shared decision-making as described in the latest ESC guideline on cardiomyopathies.^1,23,24^

**Table 5:** Clinical factors guiding the choice of serial screening interval in genotype-positive family members

	Factors supporting annual follow-up	Factors supporting 3-year follow-up
Age of family member	<40 years	>60 years
Findings on cardiac screening	Presence of structural or ECG findings^a^	Absence of structural or ECG findings
Genotype^b^	*BAG3*, *LMNA*, *RBM20*, *PLN*, *FLNCtv*, *DSP*, *MYH7*	*TTN*, gene-elusive familial disease
Predominant familial presentation	Sudden cardiac death, severe heart failure	Structural abnormalities
Age of first presentation in the family	<5 years of age in relation to affected family member	>10 years of age in relation with affected family member

Examples:
A 24-year-old male with no structural or electrocardiogram findings at screening, presence of a truncating *FLNC* variant, and sudden cardiac death in the family at age 28: annual screening. A 48-year-old female with structural or electrocardiogram findings at screening, presence of a truncating *TTN* variant, and structural abnormalities (dilated cardiomyopathy) in the family at age 40: 3-year follow-up. A 56-year-old male with no structural or electrocardiogram findings at screening, presence of *LMNA* variant and structural abnormalities (dilated cardiomyopathy) in the family at age 32: 3-year follow-up. A 35-year-old female with late gadolinium enhancement on cardiac magnetic resonance, presence of *DSP* variant and structural abnormalities (dilated cardiomyopathy) in the family at age 24: annual screening. A 46-year-old male with T-wave inversion and low voltages on electrocardiogram at screening, presence of *MYH7* variant and structural abnormalities (dilated cardiomyopathy) in the family at age 28: annual follow-up.

CMR, cardiac magnetic resonance; DCM, dilated cardiomyopathy; ECG, electrocardiogram; LGE, late gadolinium enhancement.

^a^As defined in *Table 1*.

^b^List of genes limited to genes with prevalent pathogenic/likely pathogenic variants and definitive gene–disease association.

## Genotype–phenotype associations

Until recently, overall recommendations have not been gene- or variant-specific. However, genetic and phenotypic heterogeneity are particularly evident in DCM.^34–36^ Whereas the clinical risk of heart failure events can be predicted by the assessment of severe systolic dysfunction, arrhythmic events may occur when systolic function is still normal.^37^ A genotype-first approach could therefore provide an alternative strategy for a more precise therapeutic and prognostic management of probands with DCM, even when the phenotypes appear similar.^38,39^

### Genes with a high risk for arrhythmic events

Certain genes are characterized by higher risk of arrhythmic events irrespective of systolic dysfunction, whereas other genes are more prone to heart failure events with lower arrhythmic risk, more commonly associated with severe systolic dysfunction. The high-risk genes for arrhythmias are listed in the latest guidelines and (currently) include *LMNA*, *TMEM43*, *PLN*, *DSP*, *FLNC* (truncating variants), and *RBM20*.^1,37^ These are also genes that can lead to a more arrhythmogenic phenotype, even when the proband has a DCM phenotype. For *LMNA*, male sex, truncating variants, conduction disease, and non-sustained ventricular tachycardia identify patients at high arrhythmic risk even in the absence of severe left systolic dysfunction.^40,41^ Atrioventricular blocks or atrial arrhythmias, such as atrial fibrillation, could be the first manifestation in carriers of *LMNA* variants and be associated with higher disease penetrance and progression.^22^ Additionally, isolated atrial fibrillation could be an expression of TTNtv.^42^ Following the clinical characterization of *LMNA*, many other genes have been identified to have a pro-arrhythmic clinical course or being associated with an arrhythmogenic phenotype. These genes include *FLNC* (truncating variants),^43^  *DSP*,^25,44^  *PLN*,^45^  *TMEM43*,^46^  *EMD*,^47^  *DES*,^48^ and *RBM20*.^49^

### Role of cardiac magnetic resonance in genetic dilated cardiomyopathy

Cardiac magnetic resonance has significantly improved our understanding of the underlying causative mechanisms behind an arrhythmic phenotype.^50^ Myocardial fibrosis displaying a diffuse distribution with a ring-like pattern has been observed in cases with variants in *DSP* and *FLNC*.^43,44,51^ Notably, a typical inferolateral subepicardial LGE could be detected in carriers of arrhythmic gene variants with a preserved systolic function, representing an early feature of the disease that will be missed when screening only with echocardiography.^43–45^ Moreover, LGE predicts an elevated risk of ventricular arrhythmias, even in the presence of mildly reduced LVEF.^52,53^ Abnormal ECG findings, such as low voltages or inverted T-waves inferolaterally, are typically detected in these carriers at risk.^43–45^ Additionally, *DSP* (and less commonly, *FLNC* and *PLN*) variants could manifest through single or recurrent myocardial injury events accompanied by chest pain and troponin elevation, referred to as ‘hot phases’.^54,55^

### Genes with a predominant heart failure disease course

*TTN*-, *BAG3*-, and *MYH7*-associated DCMs follow a less arrhythmogenic and more heart failure-prone clinical course.^21,23,27^ The management of mild forms of *TTN*-, *BAG3*-, and *MYH7*-associated DCM follows the regular treatment for patients with heart failure,^56^ whereas the detection of a genetic variant in a pro-arrhythmogenic gene requires an increase in arrhythmia monitoring independent of LVEF values. In such cases, a thorough clinical evaluation and arrhythmic stratification, extending beyond systolic function assessment, become imperative.

## Innovations in family screening and future outlook

Data on the yield of early detection of cardiac disease in family members are scarce, and most recommendations in available guidelines are strongly based on expert opinion. There is still much potential in more personalized screening recommendations. Additionally, screening allows early detection of disease in family members, but treatment is currently only started when a phenotype has developed. Cascade genetic testing to guide clinical surveillance of asymptomatic family members of patients with DCM is very likely to be cost-effective, but studies investigating the cost–benefit ratio are scarce.^57^ The following sections summarize the current state of innovative advances in family screening, the current evidence, and knowledge gaps (*Table 6*).

**Table 6:** Future outlook of family screening in patients with dilated cardiomyopathy

	Potential benefits	Current evidence	Clinical practice	Gaps
Integrating genotype–phenotype	Gene-specific (and P/LP variant-specific) screening recommendations: thereby improving the sensitivity of family screening	Extensive data on genotype–phenotype associations in patients with DCM	Guideline recommendations are similar for all gene variant carriers	Scarcity of studies designed for follow-up of asymptomatic family members Limited information available regarding the gene-specific progression of the disease over time No studies investigating the potential benefit of gene-specific recommendations for family screening
Innovative diagnostics	Detection of subtle early markers of disease: preventing overt disease presentation and initiating early treatment	GLS and AI-ECG can detect early disease markers of DCM (and genotype-specific subgroups)	GLS is sporadically measured. Further no implementation	RCTs investigating treatment decisions based on GLS are lacking No data on AI-ECG for early disease in family members
Polygenic risk scores	More accurate risk stratification among asymptomatic family members	Risk score developed for LVESVi and applied to DCM	No implementation	No validated PRS for clinical use available yet No data on influence of non-Mendelian genetics on risk stratification of family members (with or without a P/LP variant)
Early treatment	Prevention or slowing of disease development and progression	Evidence mostly available from mouse models and underpowered trials	ACEi for children carrying a *DMD* variant	Insufficient follow-up time to observe the effect of therapy on phenotype development Limited sample size in available RCTs

NA, not applicable; DCM, dilated cardiomyopathy; ACEi, angiotensin-converting enzyme inhibitor; P/LP, pathogenic/likely pathogenic; PRS, polygenic risk score; RCT, randomized controlled trial; GLS, global longitudinal strain; AI-ECG, artificial intelligence-guided electrocardiography; LVESVi, left ventricular end-systolic volume index.

### Gene-specific screening recommendations

The disease penetrance, clinical course, and (pro-arrhythmogenic) phenotype of specific genes has become more apparent in the last decade (*Table 3*).^21–23,25–29,58–60^ However, all asymptomatic family members who carry a P/LP variant receive (roughly) comparable clinical screening recommendations, irrespective of the specific gene affected. It seems appropriate to take the specific genotype (or even P/LP variant) into account when tailoring dedicated, gene-oriented, cardiac screening in family members.

On average, the onset of cardiac disease in genetic DCM is around 40 years, with *MYH7*, *DSP*, *BAG3*, *RBM20*, and *LMNA* presenting at a younger age and *PLN*, *FLNC*, and *TTN* at an older age (*Table 3*).^19,21–25,27–29,43,45,59,61^ However, for *MYH7*, it is common that DCM might appear before the age of 12 years.^24^ There are certain genotypes such as *TTN* where screening intervals could probably be safely extended to 3–5 years in relatives younger than 40 years, who do not exhibit abnormalities in ECG and cardiac imaging tests. There are distinct sex differences in penetrance, most pronounced in *TTN*, *MYH7*, *LMNA*, and *RBM20*, where the penetrance of disease in females is delayed by ∼10 years. Notably, in patients with *DSP*, penetrance seems to be higher in females, and patients may present with myocarditis-like symptoms.^25,44^ In the future, these data can eventually be included in personalizing screening recommendations for family members. Future studies should include genotype-positive family members and aim to determine the gene-specific clinical predictors for phenotype development, comparable to the current trend of gene-specific risk calculation for major arrhythmic events and sudden cardiac death in overt disease.

Arrhythmias can be the first presentation in patients with P/LP variants in *LMNA*, *RBM20*, *FLNCtv*, *DES*, *DSP*, and *PLN*, before signs of systolic dysfunction are visible (i.e. presenting with an arrhythmogenic phenotype, often without LV dilatation). It can be considered to screen asymptomatic carriers of these genes on a yearly basis with ECG, echo, and ambulatory ECG monitoring even when there are no structural abnormalities. The presence of LGE on CMR is prevalent in patients with DCM when there is an underlying genetic aetiology, although this is found rarely in *MYH7*, *RBM20*, and TNNT2.^20^ In addition, patterns of LGE can point towards certain likely causative genes (ring-like, subepicardial vs mid-wall patterns).^20^ Screening with CMR is advised in genotype-positive family members, but especially in those with *FLNC*, *PLN*, and *DSP* variants (i.e. genes associated with a high-risk arrhythmic phenotype). Eventually, a risk prediction model, including genetic and non-genetic factors to determine risk on DCM for an individual, would facilitate individualized decision-making on prevention and screening, as it is already available for breast cancer.^62^

### Innovating diagnostics for the detection of early disease in family members

Novel diagnostic modalities that can detect very subtle cardiac abnormalities that could be signs of early disease are emerging. Artificial intelligence (AI)-guided ECG analysis is able to detect signs on an ECG that are specific for carries of genetic variants (e.g. *PLN*) and could guide future screening of family members in those healthcare settings that have limited resources or facilities.^63^ Additionally, AI-ECG is also able to detect structural abnormalities of the heart.^64–68^ Therefore, AI-ECG analysis has great potential to become a first-line screening tool for both arrhythmias and cardiac dysfunction in family members. Whether the use of AI-ECG has the desired sensitivity and specificity to lead to an earlier detection compared with the current follow-up strategy in routine care still needs to be determined. The use of AI-ECG also carries the possibility to be translated towards portable and wearable devices providing the opportunity for remote monitoring in this patient category to reduce in-person follow-up and unnecessary diagnostic screening.^69^

The use of echocardiographic tissue deformation imaging (especially global longitudinal strain analysis) has the potential as a sensitive screening tool for early detection of genetic cardiomyopathies in family members.^70–72^ Better understanding of its sensitivity, specificity, and accuracy is still required, as well as insights into how early changes can be detected. Standardization of strain measurement and clear cut-off values independent of used vendor will be required before clinical implementation. In addition, consensus about the therapeutic implications of an abnormal strain is still lacking. Two possible scenarios exist: (i) shorter cardiac screening intervals or (ii) start of heart failure therapy. Waiting until the LVEF decreases to initiate treatment could miss a valuable opportunity for treatment in the early disease stage. Besides echocardiographic deformation imaging, new echocardiographic modalities such as shear wave elastography are upcoming, with great potential as screenings tool.^73^ Future studies will need to focus on these clinical scenarios before advancing towards clinical implementation.

### Potential value of non-Mendelian genetic factors in risk prediction

Genetic testing in current clinical practice aims to identify monogenic forms of DCM, which is detected in up to 20%–30%.^2,74^ The 70%–80% genotype-negative/elusive patients probably constitutes a group where other Mendelian genes are yet to be discovered, or a more complex genetic architecture combined with non-genetic factors might play a pivotal role.^75^ The genetic and environmental interaction might also explain in part the incomplete penetrance and differences in disease severity observed in families.^76,77^

The absence of a monogenic aetiology in a patient with DCM does not exclude heritability of the disease; i.e. there is still an increased risk of DCM for (first-degree) family members.^33,78^ Therefore, family members are still recommended for serial clinical screening according to the 2023 ESC cardiomyopathy guideline,^1^ although their risk is probably lower compared with families where the family members carry a P/LP variant (*Table 4*). For families in whom genetic testing revealed no P/LP variant, identification of non-Mendelian genetic factors that collectively lead to DCM may have the potential to better identify those family members at high risk, who will benefit from clinical screening, and to reassure those family members at low risk. Also, family members carrying a P/LP variant, who have a favourable genetic background, may be less likely to develop DCM. This is an active field of research, but there is currently insufficient evidence that identification of multiple intermediate or low effect variants explains the inheritability of DCM within a family, or that it might influence risk stratification in family members.

Polygenic risk scores (PRSs) represent the sum of multiple single common variants that all carry a small disease risk individually. In contrast to one single P/LP variant that carries a high risk for developing DCM, it is a measure calculated by aggregation of variants conferring small risks. There are few PRSs published in relation to DCM and related LV traits such as the end-systolic volume.^76,77,79^ Polygenic risk scores have been associated with the incidence of DCM in the population, as it also affects LV volume and function in those carrying a pathogenic variant in a DCM-associated gene. To date, the clinical value and applicability of a PRS have not been demonstrated. Future studies should validate the contribution of PRS in the risk stratification of family members of a patient with DCM, both in the presence and absence of a P/LP variant.

### Lifestyle advice and early treatment to prevent disease development

Lifestyle recommendations are relevant for asymptomatic carriers of a P/LP variant. With respect to exercise, the latest guidelines recommend avoiding moderate- and high-intensity exercise for carriers of a P/LP variant in *LMNA*, *TMEM43*, or *FLNC*, as exercise may have an adverse effect on cardiac function and risk of potentially fatal arrhythmias.^1,80^ For phospholamban (PLN), it was shown that there is no effect of exercise on disease penetrance in asymptomatic variant carriers.^81^ Female family members with P/LP variants can become pregnant in a normal way and have uneventful pregnancies, but development of DCM during pregnancy has been reported in a subset of asymptomatic genetic carriers.^82^ Female patients with LMNA variants may experience increased arrhythmias during pregnancy, but adverse electrical or structural long-term outcome after pregnancies was not shown.^83^ Genetic studies in patients with peripartum cardiomyopathy (PPCM) have revealed genetic similarity with DCM.^84,85^ Therefore, cardiac evaluation during pregnancy and after delivery seems appropriate and can be discussed with female carriers of a P/LP variant without a phenotype. Comparable genetic overlap with other forms of cardiomyopathy have been shown (e.g. alcoholic-induced cardiomyopathy), and therefore carriers of a P/LP variant should therefore be encouraged to avoid excessive alcohol intake.^86^

It has been suggested that the beneficial effects of commonly used heart failure medications could be extended to subjects at risk of developing heart disease, such as family members carrying a P/LP variant, or other settings. For example, the usefulness of angiotensin-converting enzyme inhibitors (ACEis) and angiotensin II receptor blockers (ARBs) in the prevention of ventricular dysfunction in patients undergoing chemotherapy with anthracyclines has been demonstrated in double-blind controlled trials and several meta-analyses.^87,88^ Further evidence that treatment with inhibitors of the renin–angiotensin–aldosterone system might prevent the progression of ventricular dysfunction comes from animal models. A mouse model with DCM due to a known pathogenic variant in *TNNT2* (p.Lys210del) was treated preventively with candesartan, which led to a dramatic improvement in survival compared with the untreated mice.^89^ In addition, myocardial function was also improved and was similar to that found in wild-type mice. Preventive treatment with enalapril in an over-expressing transgenic mouse of the p.(Ser358Leu) mutation in *TMEM43* increased survival, reduced cardiac fibrosis, and slowed deterioration of cardiac function compared with their untreated littermates.^90^

In humans, the benefits of early treatment in genetic carriers without overt cardiac disease are very limited and restricted currently to patients with Duchenne muscular dystrophy (DMD).^91^ Preventive treatment with perindopril is nowadays advised in male patients with DMD to prevent DCM onset based on a multi-centre, randomized, double-blind trial of 56 children with DMD who were treated with perindopril or placebo for 3 years and then with open-label perindopril for additional 2 years.^91^ After the initial 3 years, there were no significant differences across groups, but after the 5-year follow-up, only one patient in the group who received continuous perindopril developed DCM compared with eight subjects in the delayed perindopril group. Moreover, other small studies in children with DMD have suggested that the addition of eplerenone to background ACEi or ARB therapy attenuates the progressive decline in LV systolic function.^92^

Unfortunately, trials with preventive therapies in healthy individuals with other DCM-causing variants had many difficulties. A double-blinded placebo-controlled European study evaluating perindopril in family members carrying a DCM-causing variant [the *INtegrated HEart Research In TrANslational genetics of dilated Cardiomyopathies in Europe* (INHERITANCE) project] was prematurely stopped in 2013 due to low enrolment. A recently published study evaluating eplerenone in *PLN* (p.Arg14del) variant carriers was underpowered to see an effect after 3 years of follow-up [the *PHOspholamban RElated CArdiomyopathy intervention STudy* (i-PHORECAST)].^61^ Of note, a multi-centre, international, double-blinded, placebo-controlled trial is currently evaluating the effect of candesartan in preventing DCM development among 320 healthy carriers of DCM-associated variants in the absence of a phenotype (the EARLY-GENE trial, NCT05321875). Results of this trial are highly awaited, although the planned 3-year duration and limited sample size may not allow for statistically significant differences across groups. Currently, routine use of preventive treatment with any heart failure medication does not seem appropriate for genetic carriers of DCM-causing variants, with the exception of children with DMD.

## Summary

In this clinical consensus statement, we describe the current guideline recommendations for clinical and genetic screening of family members of patients with DCM and highlight the future opportunities to improve the care of family members. Advances in geno- and phenotyping and the detection of intermediate- and low-risk variants have the potential to increase the risk stratification in family members, subsequently leading to a more personalized approach. It will be very important to focus on (i) education of cardiologists in genetics and genomics, (ii) specialized centres, and (iii) local, national, and international registries with follow-up and precise outcome data of family members.

## Declarations

### Data Availability

No data were generated or analysed for this manuscript.
